# Midgut expression of immune-related genes in *Glossina palpalis gambiensis* challenged with *Trypanosoma brucei gambiense*

**DOI:** 10.3389/fmicb.2014.00609

**Published:** 2014-11-10

**Authors:** Illiassou Hamidou Soumana, Bernadette Tchicaya, Paul Chuchana, Anne Geiger

**Affiliations:** ^1^IRD-CIRAD, UMR 177Montpellier, France; ^2^Inserm, U844, Hôpital Saint-EloiMontpellier, France

**Keywords:** *Glossina palpalis gambiensis*, *Trypanosoma brucei gambiense* infection, midgut, immune gene expression

## Abstract

Tsetse flies from the subspecies *Glossina morsitans morsitans* and *Glossina palpalis gambiensis*, respectively, transmit *Trypanosoma brucei rhodesiense* and *Trypanosoma brucei gambiense*. The former causes the acute form of sleeping sickness, and the latter provokes the chronic form. Although several articles have reported *G. m. morsitans* gene expression following trypanosome infection, no comparable investigation has been performed for *G. p. gambiensis*. This report presents results on the differential expression of immune-related genes in *G. p. gambiensis* challenged with *T. b. gambiense*. The aim was to characterize transcriptomic events occurring in the tsetse gut during the parasite establishment step, which is the crucial first step in the parasite development cycle within its vector. The selected genes were chosen from those previously shown to be highly expressed in *G. m. morsitans*, to allow further comparison of gene expression in both *Glossina* species. Using quantitative PCR, genes were amplified from the dissected midguts of trypanosome-stimulated, infected, non-infected, and self-cleared flies at three sampling timepoints (3, 10, and 20 days) after a bloodmeal. At the 3-day sampling point, transferrin transcripts were significantly up-regulated in trypanosome-challenged flies versus flies fed on non-infected mice. In self-cleared flies, serpin-2 and thioredoxin peroxidase-3 transcripts were significantly up-regulated 10 days after trypanosome challenge, whereas nitric oxide synthase and chitin-binding protein transcripts were up-regulated after 20 days. Although the expression levels of the other genes were highly variable, the expression of immune-related genes in *G. p. gambiensis* appears to be a time-dependent process. The possible biological significance of these findings is discussed, and the results are compared with previous reports for *G. m. morsitans*.

## INTRODUCTION

Tsetse flies (*Glossina* sp.) are responsible for the cyclical transmission of protozoan known as trypanosomes, which are the causative agents of Human African Trypanosomiasis (HAT; or sleeping sickness) and Animal African Trypanosomiasis (or nagana) throughout sub-Saharan Africa (Simarro et al., 2003). It is estimated that 60 million people in 36 African countries are at risk of HAT (WHO, 2006). Sleeping sickness is fatal if untreated (Holmes, 2013). No vaccine is available for the mammalian host, as the variant surface glycoprotein (VSG) coating the trypanosome plasma membrane makes the development of a vaccine unlikely. Furthermore, part of this VSG composition and structure periodically varies, which in turn causes periodic antigenic variations that allow the trypanosome to escape both injected and/or natural host-produced antibodies. Finally, this coat prevents antibodies from gaining access to invariant surface molecules (MacGregor et al., 2012). To complicate matters, chemotherapy treatments have major harmful side effects and are difficult to administer (Priotto et al., 2008), and the emergence of parasite resistance has decreased the efficacy of drug treatments (Baker et al., 2013).

The fly vector, which is strictly hematophagous, acquires the parasite during a bloodmeal on an infected host, whether human or animal. To be transmitted, trypanosomes must first establish in the midgut; then they migrate to the salivary glands, where they mature into an infective metacyclic form; they are finally secreted in the saliva during a bloodmeal (Hu and Aksoy, 2006).

In ideal laboratory conditions, 40% or more of challenged flies will eliminate their ingested trypanosomes (Lehane et al., 2003, 2008). For field flies, infection rates rarely exceed 10% of the population (Frézil and Cuisance, 1994). Only a small number of flies are able to transmit parasites to a host (Aksoy et al., 2003; Rio et al., 2004). Approximately 72 h following ingestion of the infected bloodmeal, a process of attrition leads to the complete elimination of the infection in a high proportion of flies, whereas parasites are established in the gut during a successful infection. Flies can be arranged into two groups following this attrition process: those that are susceptible to infection when trypanosomes are detectable in the fly’s gut, and those that are refractory (or self-cleared) when trypanosomes are undetectable (Gibson and Bailey, 2003). Differential expression of midgut effector molecules in different tsetse species or strains may account for the variability in susceptibility to trypanosomes (Haddow et al., 2005). Many other factors are involved in determining the success or failure of the infection and maturation processes (Maudlin and Welburn, 1994). These include fly sex, age, and nutritional status at the time of exposure to infectious trypanosomes (Welburn and Maudlin, 1992); antimicrobial peptides (AMPs; Hao et al., 2001; Hu and Aksoy, 2006); trypanosome-binding lectins (Maudlin and Welburn, 1988; Welburn et al., 1994); gut-associated EP protein (Chandra et al., 2004; Haines et al., 2005, 2010); and reactive oxygen species (ROS; Hao et al., 2003).

Since trypanosomes initiate their cycle within the host midgut, an improved understanding of the differential expression of immunity genes could provide opportunities to identify genes possibly involved in tsetse refractoriness, as well as those involved in active infections.

Previously, Lehane et al. (2003) reported a number of selected genes (including genes related to fly immunity) that exhibit altered expression patterns in response to trypanosome infection, during their establishment in the fly gut. These studies were conducted on insectary-maintained flies belonging to the subspecies *Glossina morsitans morsitans* (initially collected in Zimbabwe) and challenged with *Trypanosoma brucei brucei*. In east African countries, flies of the *morsitans* group transmit trypanosomes belonging to the subspecies *Trypanosoma brucei rhodesiense*, causing the acute form of sleeping sickness. Conversely, *Glossina palpalis gambiensis* (*palpalis* group) flies in West Africa transmit trypanosomes belonging to the subspecies *Trypanosoma brucei gambiense*, causing the chronic form of the disease (Hoare, 1972). We chose to investigate *G. p. gambiensis* challenged with *T. b. gambiense*, as this approach had previously not been utilized to examine this specific *Glossina*/trypanosome couple. Furthermore, our choice enables checking whether the responses of the two *Glossina* subspecies to their, respectively, transmitted trypanosome subspecies are comparable or not. We investigated the *G. p. gambiensis* response at the trypanosome invasion step, as it is determinant in whether the parasite will establish within the fly gut (i.e., flies susceptible to trypanosome infection) or if it will be eliminated (i.e., flies refractory to trypanosome infection, or self-cleared/self-cured flies). Finally, we investigated 12 immune genes selected from those previously reported to be highly over-expressed in *G. m. morsitans* challenged with *T. b. brucei* (Lehane et al., 2003).

## MATERIALS AND METHODS

### ETHICS STATEMENT

All experiments on animals were conducted according to internationally recognized guidelines. The experimental protocols were approved by the Ethics Committee on Animal Experiments and the Veterinary Department of the Centre International de Recherche Agronomique pour le Développement (CIRAD), Montpellier, France.

### *T. b. gambiense* STRAIN AND FLY INFECTION

The *T. b. gambiense* isolate S7/2/2 used for fly infections was isolated in 2002 by rodent inoculation from a HAT patient detected in the sleeping sickness focus of Bonon, Côte d’Ivoire (Ravel et al., 2006).

Female *G. p. gambiensis* tsetse flies were collected from the CIRAD Baillarguet insectary. Following adult emergence, the population was maintained in a level 2 containment insectary at 23°C and 80% relative humidity (Geiger et al., 2005). This fly colony originated from Burkina Faso, where it was first collected 40 years ago.

A *T. b. gambiense* stabilate was thawed at room temperature and 0.2 ml was injected intraperitoneally into Balb/cj mice. To monitor infection, tail blood was examined using a phase-contrast microscope at 400× magnification. Teneral flies (less than 32 h old) were fed on the abdomens of infected mice (30 flies per mouse, on average); mice displayed parasitemia levels between 16 and 64 × 10^6^ parasites/ml, as determined by the matching method (Herbert and Lumsden, 1976). Only flies that had ingested a large bloodmeal were retained for further studies. After 10-day and 20-day timepoints, anal drops were collected from flies that fed on infected mice, and their infection status was assessed. *T. b. gambiense* presence was determined by PCR of chelex-extracted anal drop DNA using the TBR1 and TBR2 primers (Moser et al., 1989). The presence of parasites in the anal drop was positive indication for midgut infection (i.e., susceptible flies). By contrast, the absence of the parasite indicated that these flies receiving an infected bloodmeal were refractory to infection. Anal drop analysis was selected in this study to determine fly contamination status since the whole midgut was later used for RNA extraction. The prevalence of midgut infection was less than 5% for 10-day flies and greater than 10% for 20-day flies, corresponding with recently recorded values from artificial infection experiments (Ravel et al., 2006; Hamidou Soumana et al., 2014). Using this procedure, flies were separated into infected and self-cured groups (i.e., flies that had ingested trypanosomes in their bloodmeal but had cleared the infection), and dissected according to the method described by Penchenier and Itard (1981). The 3-day group of flies received an infected bloodmeal and was dissected 3 days later; they were compared with 3-day flies fed on an uninfected bloodmeal, considered as control flies. Dissected tsetse fly midguts were collected (pool of seven fly guts per sample) in 400 μl of RNA later reagent and stored at -80°C until RNA extraction.

Sampling times were chosen according to a previously determined time course of susceptible fly infection by trypanosome (Van Den Abbeele et al., 1999; Ravel et al., 2003). The 3-day and 10-day sampling times were respectively, selected to target differentially expressed genes involved in early events associated with trypanosome entry into the midgut, and the establishment of infection. The 20-day time point was selected to target genes involved in events occurring relatively late in trypanosome infection, within its vector.

### TOTAL RNA ISOLATION

Midguts were dissected from 3-day flies (fed on either an infected or a non-infected bloodmeal), as well as from infected and self-cleared flies at 10 and 20 days after the infective bloodmeal. Each timepoint consisted of four biological replicates (seven pooled midguts). Total RNA was then extracted from each sample using Trizol reagent (Invitrogen), according to the manufacturer’s specifications. RNA integrity was assessed after extraction using agarose gel electrophoresis. RNA quality and the absence of any DNA contamination were checked on an Agilent RNA 6000 Bioanalyzer and quantified using the Agilent RNA 6000 Nano kit (Agilent Technologies).

### IMMUNITY-RELATED GENES AND QUANTITATIVE REAL-TIME PCR PRIMERS

Lehane et al. (2003) identified genes with putative immune-related functions in *G. m. morsitans* following *T. b. brucei* infection. Twelve highly up-regulated genes were chosen from this study to investigate their possible differential expression in either *G. p. gambiensis* refractory flies versus *T. b. gambiense* infected flies (10- and 20-day samples), or in trypanosome-stimulated flies versus control flies (3-day samples). Gene expression was measured by quantitative PCR using specific primer pairs (**Table 1**) designed with the PrimerBlast software (http://www.ncbi.nlm.nih.gov/tools/primer-blast/). Pairs of *G. p. gambiensis* tubulin beta-1 gene-specific primers were designed using the sequences from the *G. m. morsitans* tubulin beta-1 gene (GenBank accession number, DQ377071; Attardo et al., 2006).

**Table 1:** Primers of immunity-related genes designed for quantitative PCR.

Gene symbol	Gene name	Forward primer (5′-3′)	Reverse primer (5′-3′)	Amplicon size (bp)	Accession number
GMOY003306	EP protein	GCT-GAA-GTT-GGG-AAG-ACT-GC	AGC-TTG-CTC-GAA-AGC-TTG-AT	108	AY077716.1
GMOY010190	Transferrin	CAA-CGG-GCT-TGA-GTT-TAT-CA	GTC-CCG-AAT-TGG-AAT-GTG-TC	128	AF368908.3
GMOY001942	Chitin binding protein	TGG-TTT-TGC-CGA-TGT-TCA-TA	CAA-CCC-ATC-TCC-TCC-CAT-AA	108	DQ307192.1
GMOY002442	Serpin-1	AAG-GTG-ACC-CCG-TTG-ATG-TA	ACC-TGC-TAG-GTT-AGC-GTT-CG	123	JQ312066.1
GMOY005573	Sphinginase	TCC-GAT-ATT-CCC-AGC-GTT-AG	CAC-TTT-GAG-GTA-GCC-AAC-GAC	122	JQ308535.1
GMOY000597	Thioredoxin peroxidase-2	TAA-TTC-GTG-TGC-GGA-AGA-TG	TTG-GAA-ATG-ACT-GCC-TTG-GT	117	AY625506.1
GMOY003093	Nitric oxide synthase	GGC-TTT-TCT-TTG-GTT-GTC-GT	CGG-TGT-ATT-TGG-TTC-TCT-GGA	122	AY152725.1
GMOY002443	Serpin-2	AGA-GTC-CCG-AAG-ATT-TGC-AT	TAT-AAA-TTG-CGT-GGG-CAA-CA	137	JQ312067.1
Gmm0601	Thioredoxin peroxidase-3	TTG-CTG-TGG-TAG-GCA-AAG-AA	TTT-AAT-GCG-CTC-GCT-AAA-AGA	137	AY625502.1
GMOY003656	Serpin-4	TTC-TCC-CTT-TGC-TGT-GTG-GT	ACG-CCG-AAC-GTA-TAA-CTT-GC	122	JQ312069.1
GM-489	C1-Tetrahydrofolate synthase	TAA-TTC-CGG-TTT-CCG-TAT-TCA	CGG-CTT-CGT-GGT-AGC-TAT-GT	101	EZ422151.1
GMOY000148	*Glossina* tubulin	CCA-TTC-CCA-CGT-CTT-CAC-TT	GAC-CAT-GAC-GTG-GAT-CAC-AG	149	DQ377071

### cDNA SYNTHESIS AND QUANTITATIVE REAL-TIME PCR

Samples were treated with RNase-free TURBO DNase I (Ambion). First-strand cDNA was then synthesized from 5 μg of total RNA using random hexamers and SuperScript II Reverse-Transcriptase (Invitrogen), according to the manufacturer’s instructions. Quantitative PCR was performed in triplicate using 2 μl of cDNA on an Mx3005P QPCR System (Agilent Technologies) and using the Brilliant II SYBR Green qPCR Kit (Agilent Technologies). The *G. p. gambiensis* housekeeping gene tubulin beta-1 was used as the reference gene to calculate the normalization of the relative quantification of expression. Cycle thresholds (Ct) for each reaction were obtained using the MxPRO QPCR Software (Agilent Technologies). PCR conditions were as follows: 94°C for 5 min (1 cycle); 94°C for 45 s, 60°C for 45 s, and 72°C for 1 min (39 cycles); and 72°C for 10 min (1 cycle). The amplification efficiency was checked by the standard curve method, and melting curve analysis was performed to check PCR specificity. Relative quantification was calculated using the 2^-ΔΔC(t)^ method (Livak and Schmittgen, 2001) and was determined for a given gene with respect to the calibrator.

### STATISTICAL ANALYSIS

Quantitative PCR data was analyzed using the 2^-ΔΔC(t)^ method. Data were then normalized against the *G. p. gambiensis* tubulin gene to determine the consecutive gene expression levels between infected and self-cleared flies, or stimulated and naive flies, for three timepoints post-infective bloodmeal (3, 10, and 20 days). A separate Kruskal–Wallis test (Hollander and Wolfe, 1973) was used for each immune gene, with R statistic software (version 2.15.0) for assessing differences in transcript expression levels. The transcription level of genes was expressed as the difference in Ct values and ΔCt values between infected and self-cleared flies.

## RESULTS

Midgut transcript responses of *G. p. gambiensis* were assayed using quantitative PCR amplification of selected immune-related genes at 3, 10, and 20 days after *T. b. gambiense* challenge. Transcription analysis of the multiple timepoints following the challenge were used to access information about the temporal kinetics of gene regulation in the host midgut response against trypanosome establishment.

### TRANSCRIPT VARIATION 3 DAYS AFTER FLY TRYPANOSOME CHALLENGE

**FIGURE 1:**
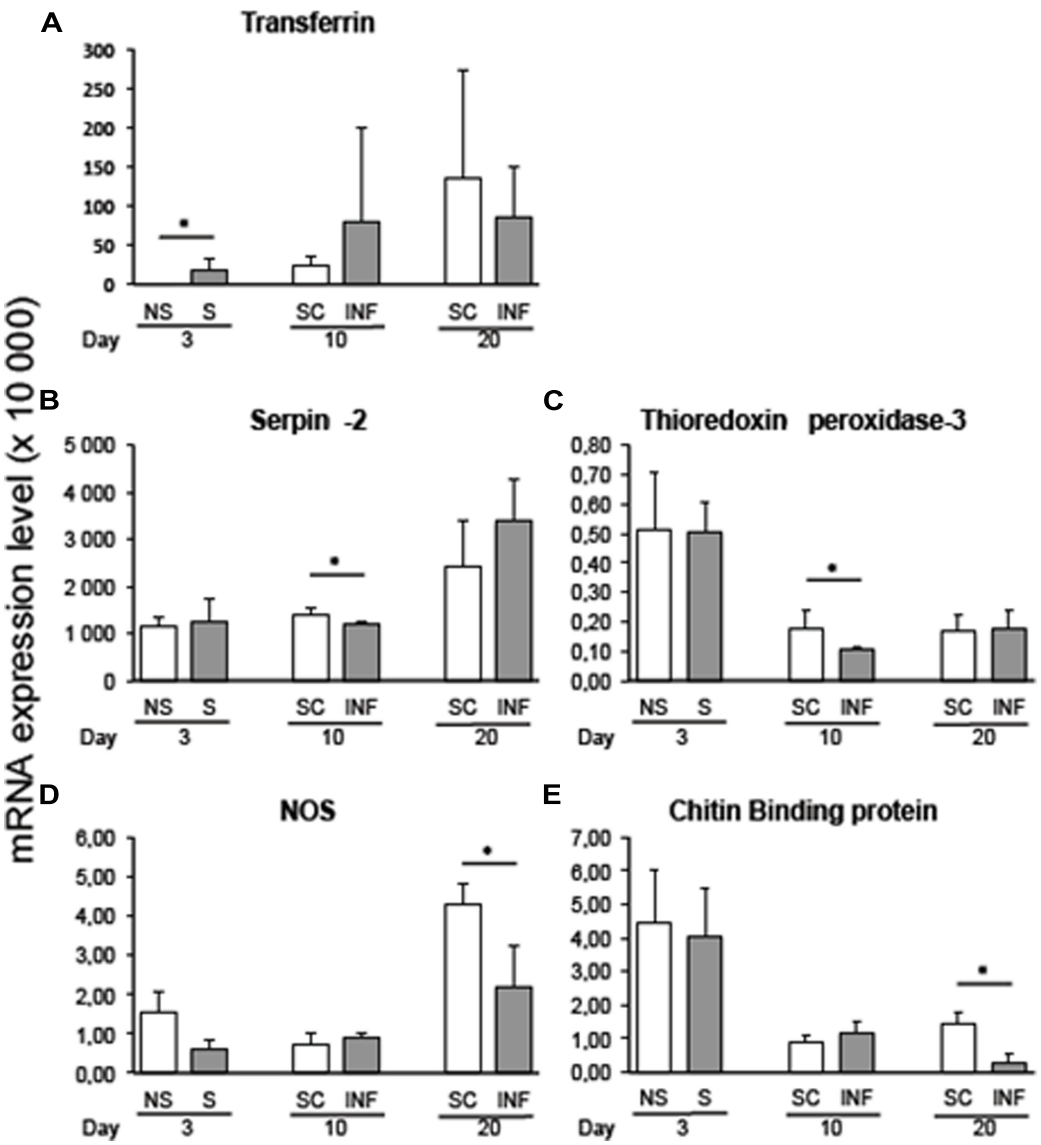
**qRT-PCR expression analysis of immune-related *Glossina palpalis gambiensis* genes at 3, 10, and 20 days post-challenge with *Trypanosoma brucei gambiense*, normalized against the *G. p. gambiensis* tubulin gene. (A)** Transferrin; **(B)** Serpin-2; **(C)** Thioredoxin peroxidase-3; **(D)** NOS; **(E)** Chitin binding protein. The “^∗^” represents significant difference between infected and self-cleared samples (*p* < 0.05).

Variation in transcript expression level was assessed at the early stage of infection by comparing 3-day flies that received an infective bloodmeal with 3-day flies fed on a non-infected bloodmeal. The infected bloodmeal induced a significant increase (*p* < 0.05) in transferrin transcripts 3 days after the trypanosome challenge (**Figure 1A**).

### GENE EXPRESSION IN INFECTED VERSUS SELF-CLEARED FLIES

Comparison of infected and self-cleared flies showed that serpin-2 and thioredoxin peroxidase-3 were expressed significantly higher (*p* < 0.05) in self-cleared flies at 10 days post-challenge with the trypanosome (**Figures 1B,C**, respectively). At 20 days post-infected bloodmeal, nitric oxide synthase (NOS) and chitin-binding protein were significantly overexpressed in refractory tsetse flies versus infected flies (*p* < 0.05; **Figures 1D,E**, respectively). Most of the other selected genes displayed differences in gene expression between refractory and infected flies, although their recorded differences were not statistically significant.

### EXPRESSION LEVEL IN SUSCEPTIBLE FLIES THROUGHOUT THE COURSE OF THE INFECTION

By comparing transcript levels at the three timepoints post-challenge with the parasite, we observed a decrease in the expression of chitin-binding protein transcripts along the progression of the infection (*p* = 0.03) for stimulated flies (3-days sampling timepoint) as compared to infected flies sampled 10 days post-infected bloodmeal uptake. Similar results were recorded for the chitin-binding protein transcript expression level, when comparing tsetse flies infected for 10 and 20 days (*p* = 0.02).

## DISCUSSION

In the present study we investigated the expression profile of immune-related genes in *G. p. gambiensis* following a *T. b. gambiense* challenge. The expression level of selected genes was compared at three crucial time points of the infection process using quantitative PCR.

Twelve immune-related genes were selected on the basis of their high differential expression in the *G. m. morsitans*/*T. b. brucei* couple, as previously reported by Lehane et al. (2003). In the *G. p. gambiensis*/*T. b. gambiense* system, only 5 of these 12 genes displayed different expression profiles between trypanosome-challenged flies and control flies.

Nitric oxide is a signaling and immune effector molecule synthesized by the NOS (Bayne et al., 2001; Rivero, 2006). NOS production is induced in *Drosophila* midgut and hemocytes challenged with bacteria or parasitoids; in mosquito it was described as a midgut-associated parasite antagonist that kills *Plasmodium* ookinetes (Peterson et al., 2007). Our results, showing a significant up-regulation of NOS transcripts in self-cleared flies at 20 days, are in agreement with those previous findings. Nitric oxide could be a part of the process leading to *T. b. gambiense* clearing in *G. p. gambiensis*. This has been demonstrated by injecting a specific NOS inhibitor into *Drosophila* body cavity prior to infection, which significantly increased parasite survival (Carton et al., 2009). However, Hao et al. (2003) showed NOS to be down-regulated by infection and not modulated by tsetse age. Its host immune response involvement may depend on the tsetse/trypanosome species couple.

Digestion of the bloodmeal can also generate ROS, which may cause damages such as enzyme inactivation, DNA degradation, and deterioration of the cellular membrane (Droge, 2002). No difference was found in the expression of thioredoxin peroxidase-2 between infected and self-cleared flies, while thioredoxin peroxidase-3 was significantly up-regulated in 10-day self-cleared flies. This enzyme may offer protection against ROS generated during the immune response (Lehane et al., 2003).

Among the three serpin (serine protease inhibitor) genes investigated, only serpin-2 was significantly over-expressed in self-cleared flies at 10 days post-challenge. The main molecular functions attributed to serpins range from the inhibition of blood coagulation to host inflammation and platelet aggregation, which are likely crucial for blood-feeding insects (Stark and James, 1995; Chmelar et al., 2011). Immune-related CLIP domain serine proteases and their inhibitors, the serpins were previously identified in *G. morsitans* (Mwangi et al., 2011). Serine proteases play an important role in the activation of the Toll or IMD pathways. Many serine proteases involved in the immune response exist in a fine balance with serine protease inhibitors to ensure that the impact of protease-activated cascades remains localized in time and space (Muta and Iwanaga, 1996; Jiang and Kanost, 2000). *Drosophila* serpin also plays a role in the regulation of Toll-mediated antifungal defense (Levashina et al., 1999; Ahmad et al., 2009). The large number of serpin transcripts found in the tsetse midgut may reflect the need to inactivate the complement and coagulation cascades of the bloodmeal, so as to protect the midgut epithelium and retain the meal in a physical state suitable for digestion. Thus, differences in serpin gene expression between the different groups of *G. p. gambiensis* flies fed on blood may reflect differences in their function according to the flies’ status.

Surprisingly, no significant changes were found in *G. p. gambiensis* EP protein transcript levels at any stage of the *T. b. gambiense* infection. In *G. m. morsitans,* EP protein was strongly up-regulated following fly challenge with Gram-negative bacteria, as well as in response to trypanosome infection (Haines et al., 2005, 2010). Furthermore, tsetse EP protein may be involved in immune modulation, as RNAi knockdown increased susceptibility to trypanosome infection. Tsetse EP protein transcript levels are, however, dramatically reduced after 3 days of starvation (Haines et al., 2010). In our study, flies were starved for 3 days prior to dissection to remove any bloodmeal in the fly gut, which could explain the absence of variation in EP protein transcripts. Akoda et al. (2009), however, reported starvation to result in a significant reduction in non-induced baseline immune gene expression, but only after a longer starvation (4 days for newly emerged flies; 7 days for older flies).

In hematophagous insects, iron-binding protein is essential for sequestering iron, which overabundance can quickly lead to oxidative stress, a potentially destructive process for membranes, proteins, and nucleic acids. The transferrin gene expression level was significantly increased in 3-day stimulated flies versus control flies. This observation is in agreement with results reported on transferrin transcription in mosquito (Yoshiga et al., 1997) and in *Bombyx mori* (Yun et al., 1999). In tsetse flies and other insects, transferrin plays multiple physiological roles in immunity, iron metabolism, and reproduction, and displays tissue-dependent expression levels (Nichol et al., 2002). As shown in other insects, transferrin mRNA levels increase upon bacterial challenge in tsetse, suggesting that transferrin may play an additional role in immunity (Guz et al., 2007). In contrast, tsetse flies that had cleared the trypanosome did not show any difference in transferrin transcript levels when compared with infected flies at 10 and 20 days post-infected bloodmeal. Similar results were reported by Lehane et al. (2003) for *T. b. brucei* infected *G. m. morsitans* versus self-cleared flies. The parasite, competing in limited dietary iron environment, may modulate host gene expression.

Chitin-binding protein gene expression increased significantly in self-cleared flies 20 days after the infected bloodmeal. Chitin is the main constituent of the peritrophic membrane (PM), a physical barrier preventing trypanosome entry into the ecto-peritrophic space, and thus constitutes an obstacle to parasite establishment in the midgut. This chitin-binding gene is homologous to the *Drosophila* gene *chit*, which encodes a chitinase-like protein (Kawamura et al., 1999). In *Sodalis glossinidius*, the secondary symbiont of the tsetse fly that favors fly infection by trypanosomes, a homologous gene encodes a chitinase that was previously hypothesized to hydrolyze pupal chitin into glucosamine which inhibits the fly midgut lectin lethal to procyclic forms of the trypanosome (Welburn and Maudlin, 1999). Based on the trypanosome developmental cycle within the tsetse fly, one would expect the increase in chitinase gene transcripts to occur much earlier than the observed 20 days post-infected bloodmeal. In *Anopheles*, for example, chitin-binding protein and the enzyme involved in PM formation both displayed increased expression 3–24 h after the bloodmeal in all flies analyzed, independent of their infection status (Dimopoulos et al., 1998). This does, however, raise an additional question on the actual role of this protein in *Anopheles*.

The overall results on *G. p. gambiensis* immune-related genes shows their expression to be highly dependent either on the stage of trypanosome invasion and/or the status of the fly (i.e., susceptible or refractory to trypanosome infection). The midgut response represents part of the *Glossina* defense arsenal against trypanosomes. Nevertheless, important variability between individual tsetse fly responses to trypanosome infection was observed. This variability could be due to variation in the size of the infected bloodmeal, and in turn to the differences in the number of ingested parasites; it could also be due to normal biological variability in the individual host’s response. In addition, significant differences were noticed between the *G. p. gambiensis* gut immune-related response and that displayed by *G. m. morsitans* following infection of the gut with *T. b. brucei*. These results strongly encourage broader investigations aimed at evaluating and identifying the factors causing these differences between the *G. p. gambiensis* and *G. m. morsitans* responses. Improved understanding in this domain is expected to be particularly relevant to identify common gene targets that would be suitable for controlling both forms of sleeping sickness. Transcriptional analysis is expected to provide data that are at the basis of the physiological response(s) of an organism to any perturbation. The recorded data will, in turn, provide further research directions that could consist, in a next step, in a proteomic analysis to assess, whether or not, the expressed genes are really translated into the corresponding proteins, and, finally, which role they actually play.

## Conflict of Interest Statement

The authors declare that the research was conducted in the absence of any commercial or financial relationships that could be construed as a potential conflict of interest.

## References

[B1] Ahmad R. Rasheed Z. Ahsan H. (2009). Biochemical and cellular toxicology of peroxynitrite: implications in cell death and autoimmune phenomenon. *Immun. Immunotoxicol.* 31 388–396 10.1080/08923970802709197 19555204

[B2] Akoda K. Van den Bossche P. Marcotty T. Kubi C. Coosemans M. De Deken R. (2009). Nutritional stress affects the tsetse fly’s immune gene expression. *Med. Vet. Entomol.* 23 195–201 10.1111/j.1365-2915.2009.00799.x 19712150

[B3] Aksoy S. Gibson W. C. Lehane M. J. (2003). Interactions between tsetse and trypanosomes with implications for the control of trypanosomiasis. *Adv. Parasitol.* 53 1–83 10.1016/S0065-308X(03)53002-0 14587696

[B4] Attardo G. M. Strickler-Dinglasan P. Perkin S. A. Caler E. Bonaldo M. F. Soares M. B. (2006). Analysis of fat body transcriptome from the adult tsetse fly, *Glossina morsitans morsitans*. *Insect Mol. Biol.* 15 411–424 10.1111/j.1365-2583.2006.00649.x 16907828

[B5] Baker N. de Koning H. P. Mäser P. Horn D. (2013). Drug resistance in African trypanosomiasis: the melarsoprol and pentamidine story. *Trends Parasitol.* 29 110–118 10.1016/j.pt.2012.12.005 23375541 PMC3831158

[B6] Bayne C. J. Hahn U. K. Bender R. C. (2001). Mechanisms of molluscan host resistance and of parasite strategies for survival. *Parasitology* 123 S159–S167 10.1017/S0031182001008137 11769280

[B7] Carton F. Frey F. Nappi A. J. (2009). Parasite-induced changes in Nitric Oxide levels in *Drosophila paramelanica*. *J. Parasitol.* 95 1134–1141 10.1645/GE-2091.1 19388790

[B8] Chandra M. Liniger M. Tetley L. Roditi I. Barry J. D. (2004). TsetseEP, a gut protein from the tsetse *Glossina morsitans*, is related to a major surface glycoprotein of trypanosomes transmitted by the fly and to the products of a *Drosophila* gene family. *Insect Biochem. Mol. Biol.* 34 1163–1173 10.1016/j.ibmb.2004.07.004 15522612

[B9] Chmelar J. Oliveira C. J. Rezacova P. Francischetti I. M. Kovarova Z. Pejler G. (2011). A tick salivary protein targets cathepsin G and chymase and inhibits host inflammation and platelet aggregation. *Blood* 117 736–744 10.1182/blood-2010-06-293241 20940421 PMC3031492

[B10] Dimopoulos G. Seeley D. Wolf A. Kafatos F. C. (1998). Malaria infection of the mosquito *Anopheles gambiae* activates immune-responsive genes during critical transition stages of the parasite life cycle. *EMBO J.* 17 6115–6123 10.1093/emboj/17.21.6115 9799221 PMC1170938

[B11] Droge W. (2002). Free radicals in the physiological control of cell function. *Physiol. Rev.* 82 47–96. 11773609 10.1152/physrev.00018.2001

[B12] Frézil J. L. Cuisance D. (1994). Trypanosomiasis, diseases with future: prospects and uncertainty. *Bull. Soc. Pathol. Exot.* 87 391–393. 7496207

[B13] Geiger A. Ravel S. Frutos R. Cuny G. (2005). *Sodalis glossinidius* (*Enterobacteriaceae*) and vectorial competence of *Glossina palpalis* gambiensis and *Glossina morsitans morsitans* for *Trypanosoma congolense* savannah type. *Curr. Microbiol.* 51 35–40 10.1007/s00284-005-4525-6 15942697

[B14] Gibson W. Bailey M. (2003). The development of *Trypanosoma brucei* within the tsetse fly midgut observed using green fluorescent trypanosomes. *Kinetoplastid Biol. Dis.* 2 1–13 10.1186/1475-9292-2-1 12769824 PMC156611

[B15] Guz N. Attardo G. M. Wu Y. Aksoy S. (2007). Molecular aspects of transferrin expression in the tsetse fly (*Glossina morsitans morsitans*). *J. Insect Physiol.* 53 715–23 10.1016/j.jinsphys.2007.03.013 17498733 PMC2065764

[B16] Haddow J. D. Haines L. R. Gooding R. H. Olafson R. W. Pearson T. W. (2005). Identification of midgut proteins that are differentially expressed in trypanosome-susceptible and normal tsetse flies (Glossina morsitans morsitans). *Insect Biochem. Mol. Biol.* 35 425–433 10.1016/j.ibmb.2005.01.015 15804576

[B17] Haines L. R. Jackson A. M. Lehane M. J. Thomas J. M. Yamaguchi A. Y. Haddow J. D. (2005). Increased expression of unusual EP repeat-containing proteins in the midgut of the tsetse fly (*Glossina*) after bacterial challenge. *Insect Biochem. Mol. Biol.* 35 413–423 10.1016/j.ibmb.2005.01.005 15804575

[B18] Haines L. R. Lehane S. M. Pearson T. W. Lehane M. J. (2010). Tsetse EP protein protects the fly midgut from trypanosome challenge. *PLoS Pathog.* 6:e1000793 10.1371/journal.ppat.1000793 PMC2832768 20221444

[B19] Hamidou Soumana I. Loriod B. Ravel S. Tchicaya B. Simo G. Rihet P. (2014). The transcriptional signatures of *Sodalis glossinidius* in the *Glossina palpalis gambiensis* flies negative for *Trypanosoma brucei gambiense* contrast with those of this symbiont in tsetse flies positive for the parasite: possible involvement of a *Sodalis*-hosted prophage in fly *Trypanosoma refractoriness*? *Infect Genet. Evol*. 24 41–56 10.1016/j.meegid.2014.03.005 24637266

[B20] Hao Z. Kasumba I. Aksoy S. (2003). Proventriculus (cardia) plays a crucial role in immunity in tsetse fly (Diptera: Glossinidiae). *Insect Biochem. Mol. Biol.* 33 1155–1164 10.1016/j.ibmb.2003.07.001 14563366

[B21] Hao Z. Kasumba I. Lehane M. J. Gibson W. C. Kwon J. Aksoy S. (2001). Tsetse immune responses and trypanosome transmission: implications for the development of tsetse-based strategies to reduce trypanosomiasis. *Proc. Natl. Acad. Sci. U.S.A.* 98 12648–12653 10.1073/pnas.221363798 11592981 PMC60108

[B22] Herbert W. J. Lumsden W. H. (1976). *Trypanosoma brucei*: a rapid “matching” method for estimating the host’s parasitemia. *Exp. Parasitol.* 40 427–431 10.1016/0014-4894(76)90110-7 976425

[B23] Hoare C. A. (1972). *The Trypanosomes of Mammals, a Zoological Monograph.* Oxford: Blackwell Scientific Publications.

[B24] Hollander M. Wolfe D. A. (1973). *Nonparametric Statistical Methods.* New York: John Wiley and Sons. 503.

[B25] Holmes P. (2013). Tsestetransmtted trypanosomes - their biology, disease impact and control. *J. Invertebr. Pathol.* 112 S11–S14 10.1016/j.jip.2012.07.014 22841638

[B26] Hu C. Aksoy S. (2006). Innate immune responses regulate trypanosome parasite infection of the tsetse fly Glossina morsitans morsitans. *Mol. Microbiol.* 60 1194–1204 10.1111/j.1365-2958.2006.05180.x 16689795

[B27] Jiang H. Kanost M. R. (2000). The clip-domain family of serine proteinases in arthropods. *Insect Biochem. Mol. Biol.* 30 95–105 10.1016/S0965-1748(99)00113-7 10696585

[B28] Kawamura K. Shibata T. Saget O. Peel D. Bryant P. J. (1999). A new family of growth factors produced by the fat body and active on Drosophila imaginal disc cells. *Development* 126 211–219. 9847235 10.1242/dev.126.2.211

[B29] Lehane M. J. Aksoy S. Gibson W. Kerhornou A. Berriman M. Hamilton J. (2003). Adult midgut expressed sequence tags from the tsetse fly *Glossina morsitans morsitans* and expression analysis of putative immune response genes. *Genome Biol.* 4 R63. 10.1186/gb-2003-4-10-r63 PMC328452 14519198

[B30] Lehane M. J. Gibson W. Lehane S. M. (2008). Differential expression of fat body genes in *Glossina morsitans morsitans* following infection with *Trypanosoma brucei brucei*. *Int. J. Parasitol.* 38 93–101 10.1016/j.ijpara.2007.06.004 17697681

[B31] Levashina E. A. Langley E. Green C. Gubb D. Ashburner M. Hoffmann J. A. (1999). Constitutive activation of toll-mediated antifungal defense in serpin-deficient Drosophila. *Science* 285 1917–1919 10.1126/science.285.5435.1917 10489372

[B32] Livak K. J. Schmittgen T. D. (2001). Analysis of relative gene expression data using real-time quantitative PCR and the 2-ΔΔ C(t) method. *Methods* 25 402–408 10.1006/meth.2001.1262 11846609

[B33] MacGregor P. Szöoõr B. Savill N. J. Matthews K. R. (2012). Trypanosomal immune evasion, chronicity and transmission: an elegant balancing act. *Nat. Rev. Microbiol.* 10 431–438 10.1038/nrmicro2779 22543519 PMC3834543

[B34] Maudlin I. Welburn S. C. (1988). The role of lectins and trypanosome genotype in the maturation of midgut infections in *Glossina morsitans*. *Trop. Med. Parasitol.* 39 56–58. 3387828

[B35] Maudlin I. Welburn S. C. (1994). Maturation of trypanosome infections in tsetse. *Exp. Parasitol.* 79 202–205 10.1006/expr.1994.1081 8056082

[B36] Moser D. R. Cook G. A. Ochs D. E. Bailey C. P. McKane M. R. Donelson J. E. (1989). Detection of *Trypanosoma congolense* and *Trypanosoma brucei* subspecies by DNA amplification using the polymerase chain reaction. *Parasitology* 99 57–66 10.1017/S0031182000061023 2797872

[B37] Muta T. Iwanaga S. (1996). The role of hemolymph coagulation in innate immunity. *Curr. Opin. Immunol.* 8 41–47 10.1016/S0952-7915(96)80103-8 8729445

[B38] Mwangi S. Murungi E. Jonas M. Christoffels A. (2011). Evolutionary genomics of *Glossina morsitans* immune-related CLIP domain serine proteases and serine protease inhibitors. *Infect. Genet. Evol.* 11 740–745 10.1016/j.meegid.2010.10.006 21055483

[B39] Nichol H. Law J. H. Winzerling J. J. (2002). Iron metabolism in insects. *Ann. Rev. Entomol.* 47 535–559 10.1146/annurev.ento.47.091201.145237 11729084

[B40] Penchenier L. Itard J. (1981). Une nouvelle technique de dissection rapide des glandes salivaires et de l’intestin de glossines. *Cah. O.R.S.T.O.M. Serie Ent. méd. et Parasitol.* 19 55–57.

[B41] Peterson T. M. Gow A. J. Luckhart S. (2007). Nitric oxide metabolites induced in *Anopheles stephensi* control malaria parasite infection. *Free Radical Biol. Med.* 42 132–142 10.1016/j.freeradbiomed.2006.10.037 17157200 PMC1764505

[B42] Priotto G. Pinoges L. Fursa I. B. Burke B. Nicolay N. Grillet G. (2008). Safety and effectiveness of first line eflornithine for *Trypanosoma brucei* gambiense sleeping sickness in Sudan: cohort study. *BMJ* 336 705–708 10.1136/bmj.39485.592674.BE 18321960 PMC2276259

[B43] Ravel S. Grébaut P. Cuisance D. Cuny G. (2003). Monitoring the developmental status of *Trypanosoma brucei* gambiense in the tsetse fly by means of PCR analysis of anal and saliva drops. *Acta Trop.* 88 161–165 10.1016/S0001-706X(03)00191-8 14516928

[B44] Ravel S. Patrel D. Koffi M. Jamonneau V. Cuny G. (2006). Cyclical transmission of *Trypanosoma brucei gambiense* in *Glossina palpalis gambiensis* displays great differences among field stocks isolates. *Acta Trop.* 100 151–156 10.1016/j.actatropica.2006.09.011 17069743

[B45] Rio R. V. Hu Y. Aksoy S. (2004). Strategies for the home team: symbiosis exploited for vector-borne disease control. *Trends Microbiol.* 12 325–336 10.1016/j.tim.2004.05.001 15223060

[B46] Rivero A. (2006). Nitric oxide: an antiparasitic molecule of invertebrates. *Trends Parasitol.* 22 219–225 10.1016/j.pt.2006.02.014 16545612

[B47] Simarro P. P. Louis F. J. Jannin J. (2003). Sleeping sickness, forgotten illness: what are the consequences in the field? *Med. Trop.* 63 231–235. 14579457

[B48] Stark K. R. James A. A. (1995). A factor Xa-directed anticoagulant from the salivary glands of the yellow fever mosquito *Aedes aegypti*. *Exp. Parasitol.* 81 321–331 10.1006/expr.1995.1123 7498429

[B49] Van Den Abbeele J. Claes Y. van Bockstaele D. Le Ray D. Coosemans M. (1999). *Trypanosoma brucei* spp. development in the tsetse fly: characterization of the post-mesocyclic stages in the foregut and proboscis. *Parasitology* 118 469–478 10.1017/S0031182099004217 10363280

[B50] Welburn S. C. Maudlin I. (1992). The nature of the teneral state in *Glossina* and its role in the acquisition of trypanosome infection in tsetse. *Ann. Trop. Med. Parasitol.* 86 529–536. 1288435 10.1080/00034983.1992.11812703

[B51] Welburn S. C. Maudlin I. (1999). Tsetse-trypanosome interactions: rites of passage. *Parasitol. Today* 15 399–403 10.1016/S0169-4758(99)01512-4 10481151

[B52] Welburn S. C. Maudlin I. Molyneux D. H. (1994). Midgut lectin activity and sugar specificity in teneral and fed tsetse. *Med. Vet. Entomol.* 8 81–87 10.1111/j.1365-2915.1994.tb00391.x 8161852

[B53] WHO. (2006). Human African trypanosomiasis (sleeping sickness): epidemiological update. *Wkly. Epidemiol. Rec.* 81 71–80. 16673459

[B54] Yoshiga T. Hernandez V. P. Fallon A. M. Law J. H. (1997). Mosquito transferrin, an acute-phase protein that is up-regulated upon infection. *Proc. Nat. Acad. Sci. U.S.A.* 94 12337–12342 10.1073/pnas.94.23.12337 PMC24933 9356450

[B55] Yun E. Y. Kang S. W. Hwang J. S. Goo T. W. Kim S. H. Jin B. R. (1999). Molecular cloning and characterization of a cDNA encoding a transferrin homolog from *Bombyx mori*. *Biol. Chem.* 380 1455–1459 10.1515/BC.1999.188 10661875

